# Co-occurring Acromioclavicular Joint Cyst and Hemarthrosis of the Shoulder Associated With Rotator Cuff Tear Arthropathy

**DOI:** 10.7759/cureus.23353

**Published:** 2022-03-21

**Authors:** Takanobu Higashi, Yutaka Mifune, Hanako Nishimoto, Atsuyuki Inui, Yuichi Hoshino, Takehiko Matsushita, Takahiro Niikura, Ryosuke Kuroda

**Affiliations:** 1 Department of Orthopaedic Surgery, Kobe University Graduate School of Medicine, Kobe, JPN

**Keywords:** reverse total shoulder arthroplasty, distal clavicle resection, cuff tear arthropathy, hemarthrosis of the shoulder, acromioclavicular joint cysts

## Abstract

Both acromioclavicular joint (ACJ) cysts and hemarthrosis of the shoulder are rare conditions of massive rotator cuff tear that eventually lead to cuff tear arthropathy. We herein report the first case of a patient with co-occurring ACJ cyst and hemarthrosis of the shoulder. An 80-year-old right-hand-dominant man presented to our outpatient department with a six-month history of repeatable right shoulder pain and swelling. Clinical examination revealed a 5 x 5 x 5 cm elastic hard or hard shoulder lump overlying the ACJ on skin with subcutaneous bleeding and swelling of the shoulder. Shoulder pain at rest and a fully reduced active range of motion (ROM), particularly in flexion and abduction, were also noted. Radiographs demonstrated moderate degeneration of the glenohumeral joint including a bone cyst of the humeral head. Magnetic resonance imaging (MRI) revealed a massive rotator cuff tear with atrophy of the supraspinatus, infraspinatus, and subscapularis muscles. The T2-weighted MRI images showed that the cyst was in direct contact with the markedly degenerated glenohumeral joint. Based on these findings, the patient was diagnosed with massive rotator cuff tear with ACJ cyst and hemarthrosis of the shoulder. The patient underwent distal clavicle resection and reverse total shoulder arthroplasty (RSA). At 12-month follow-up, the patient showed no pain symptoms, no recurrence of the cyst, and excellent ROM. We experienced a very rare case of ACJ cyst and hemarthrosis of the shoulder occurring simultaneously with rotator cuff tear arthropathy. This report is very valuable in that it suggests that RSA is useful for both ACJ cysts and hemarthrosis of the shoulders associated with rotator cuff tear arthropathy.

## Introduction

An acromioclavicular joint (ACJ) cyst is a rarely reported condition that may result from massive rotator cuff tears and degenerative osteoarthritis of the ACJ and glenohumeral joints [1]. It could interfere with the patient’s cosmetic appearance and activities of daily life. There is no consensus on the treatment of this disease, and only one case has been successfully treated with reverse shoulder arthroplasty (RSA) and minimal ACJ resection arthroplasty [2].

Hemarthrosis of the shoulder is also a rare condition that is caused by a massive rotator cuff tear with resultant cuff tear arthropathy. McCarty et al. published two cases and five reviews in 1994 in which rotator cuff tears were associated with recurrent bleeding [3]. Although there is also no consensus regarding the management of this condition, there is an opinion that RSA is useful for its treatment [4].

We present the first case report of a patient with co-occurring ACJ cyst and hemarthrosis of the shoulder. We treated the patient with RSA and excisions of the cyst and distal clavicle.

## Case presentation

An 80-year old right-hand-dominant man presented to the outpatient department with a six-month history of recurrent right shoulder pain and swelling, which was improved by a one-week rest of his right shoulder. He had no previous diagnosis or treatment prior to his surgery. He had a right shoulder fracture 40 years ago and underwent conservative therapy but the details surrounding the treatment were unknown. His past medical history included hypertension and subarachnoid hemorrhage. He had no episode of recent trauma and stroke. His mental state was normal.

**Figure 1:**
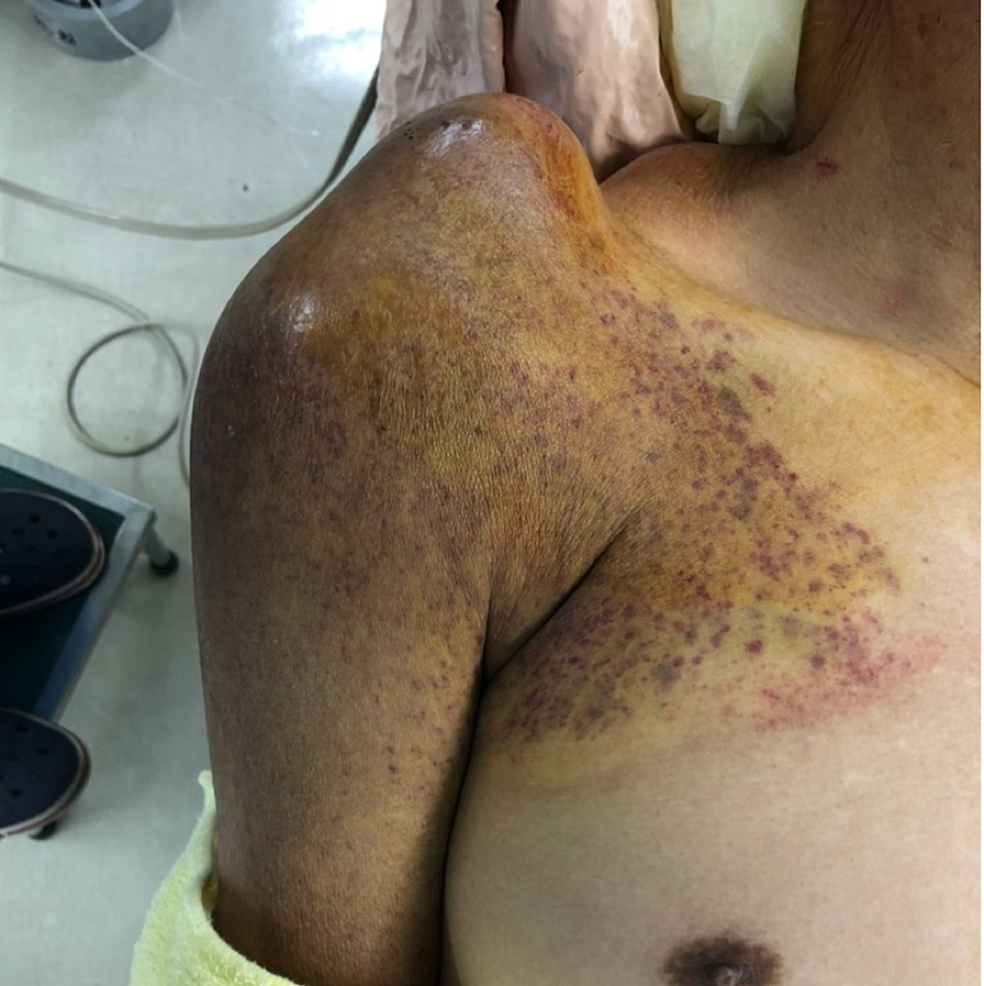
Preoperative picture A 5 x 5 x 5 cm shoulder lump overlying the acromioclavicular joint with subcutaneous bleeding.

Clinical examination demonstrated a 5 x 5 x 5 cm elastic-like or hard shoulder lump overlying the ACJ on the skin with subcutaneous bleeding and swelling of the shoulder, as well as shoulder pain at rest and a fully reduced active range of motion (ROM), particularly in flexion and abduction (forward flexion 0°, abduction 0°, external rotation 20°, internal rotation to level of the hip) (Figure 1).

**Figure 2:**
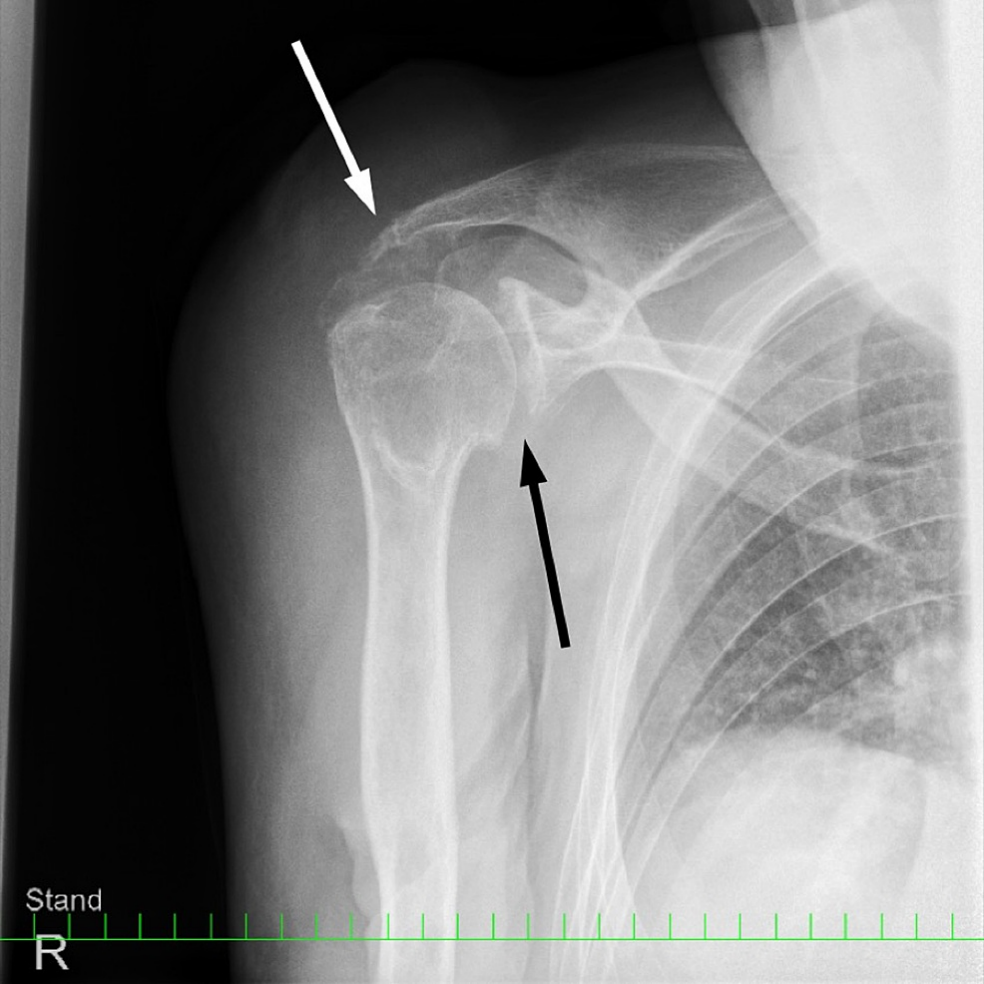
X-ray at presentation Degenerative changes in the glenohumeral joint (black arrow) and acromioclavicular joint (white arrow) with bone cysts of the humeral head.

Radiographs showed moderate degeneration of the glenohumeral joint including a bone cyst of the humeral head (Figure 2).

**Figure 3:**
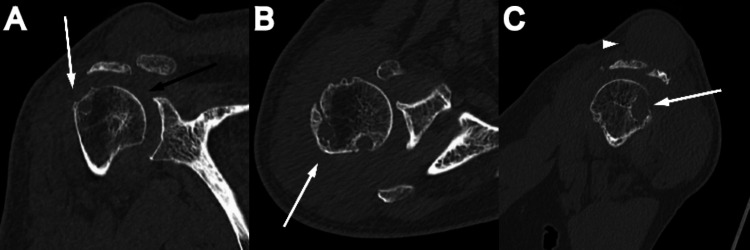
Computed tomography images Moderate degenerative changes in the acromioclavicular joint (ACJ) and glenohumeral joint (black arrow) including a bone cyst around the greater tuberosity (white arrow) (A, B, C) and a cyst projecting superior to the ACJ (arrow head) (C).

A cyst projected superior to the ACJ, with moderate degenerative changes in the ACJ and glenohumeral joint including a bone cyst around the greater tuberosity (Figure 3).

**Figure 4:**
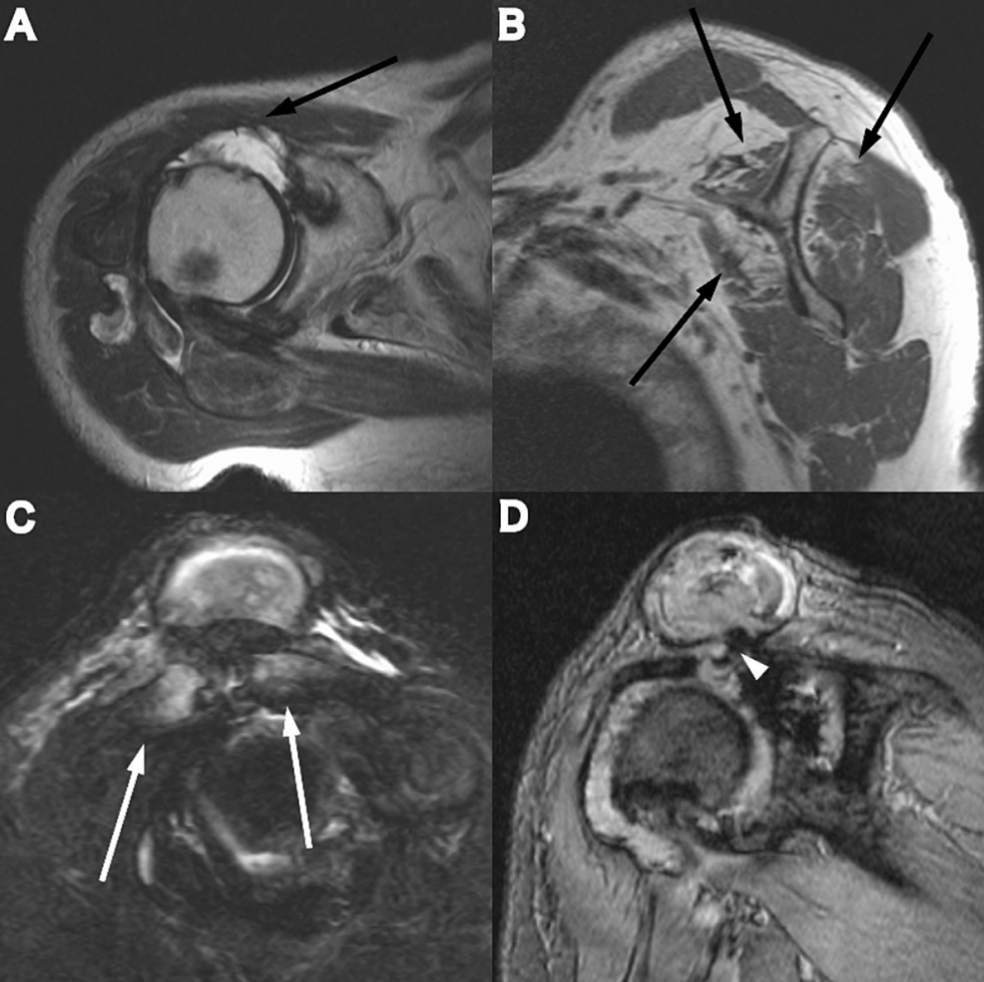
Magnetic resonance images A massive rotator cuff tear with atrophy of the supraspinatus, infraspinatus, and subscapularis muscles (black arrow) (A, B), and osteoarthritis of the acromioclavicular joint (white arrow) (C). The cyst communicated directly with the markedly degenerated glenohumeral joint (Geyser sign) on the T2-weighted MRIs (arrow head) (D).

Magnetic resonance imaging (MRI) demonstrated a massive rotator cuff tear with atrophy of the supraspinatus, infraspinatus, and subscapularis muscles. The cyst directly communicated with the markedly degenerated glenohumeral joint on the T2-weighted MRI images (Figure 4). Blood cells and fibrin clots without inflammatory and malignant cells were aspirated from the cyst, and culture of the fluid was negative.

**Figure 5:**
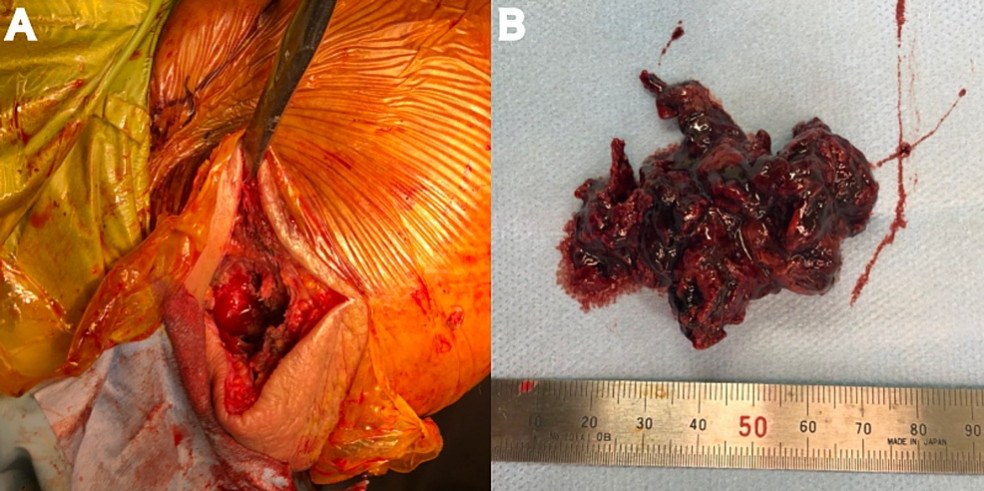
Intraoperative pictures of the right shoulder and the material for surgery When an incision was made directly over the palpable cyst, there was a massive leakage of coagulant. After the 6 x 5 cm wall of the cyst was excised as much as possible, distal clavicular excision was performed.

The patient then underwent RSA - DePuy Delta Xtend™ Reverse Shoulder System (DePuy Synthes, Warsaw, USA). Under general anesthesia, the ROM was 140° in flexion and abduction. When an incision was made directly over the palpable cyst, there was a massive leakage of coagulant. After the wall of the cyst was excised as much as possible, distal clavicular excision was performed (Figure 5).

Next, a deltopectoral approach of the shoulder was utilized for the arthroplasty. Massive rotator cuff tear and cuff tear arthropathy were detected with irreparable tears of supraspinatus, infraspinatus, and subscapularis, a partial tear of teres minor, and a rupture of the long head of the biceps tendon. The implants were inserted. Postoperatively, two-week sling fixation and rehabilitation programs were prescribed.

**Figure 6:**
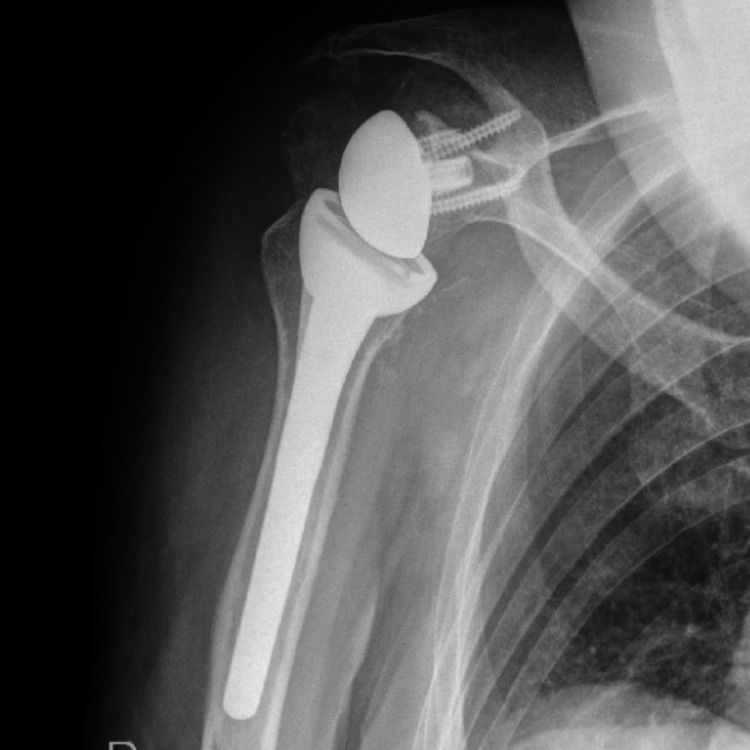
Postoperative shoulder radiograph The component of the right reverse total shoulder arthroplasty was in a satisfactory position.

A histopathological examination revealed the structural changes to be a synovial cyst. At the 12-month follow-up, the incision was well healed with remission of the cyst. The patient had no pain and had excellent ROM (forward flexion 160°, abduction 120°, external rotation 60°, and internal rotation to the level of the 4th lumbar vertebra); postoperative radiograph showed that the prosthesis was in a satisfactory position (Figure 6).

## Discussion

We experienced a very rare case of ACJ cyst and hemarthrosis of the shoulder occurring simultaneously with rotator cuff tear arthropathy. To the best of our knowledge, there is no similar report in the literature.

ACJ cysts are rare and were first described by Craig et al. in 1986 [1]. Hiller et al. classified ACJ cysts into two types according to the presence or absence of rotator cuff tears. Type 1 cysts without rotator cuff tears were formed due to damage to the ACJ alone and did not show any communication with the glenohumeral joint, while type 2 cysts were formed after arthropathic changes associated with rotator cuff tears and showed communication between the glenohumeral joint and the ACJ. The loss of function of the supraspinatus muscle results in the upward migration of the humeral head due to the unopposed action of the deltoid muscle. The elevation of the humeral head is thought to cause damage to the inferior capsule of the ACJ in the long term [5]. Synovial fluid from the glenohumeral joint escapes through the subacromial/subdeltoid bursa (Geyser sign) into the ACJ, where the degraded capsule acts like a one-way valve, preventing fluid from returning and forming cysts [6]. This case is unique in that the cyst shrank after a week of rest. We speculate that the one-week rest reduced outflow into the cyst and the hematoma was gradually absorbed because subcutaneous bleeding around his right shoulder was seen at the time of the initial diagnosis. In cases of ACJ cyst where the associated rotator cuff tear is irreparable, ACJ excision arthroplasty should be performed with adequate resection of the neck of the cyst. This removes the pinch valve effect and prevents future recurrence [2,7]. According to the review by Christodoulou et al., cystectomy, distal clavicle resection, ACJ resection, artificial head replacement, RSA, rotator cuff repair, and arthroscopic surgery have been performed for ACJ cyst [8]. However, only one case of RSA has been reported [2]. The case reported in the present study was classified as type 2 and underwent distal clavicle resection to remove the pinch valve structure. RSA was chosen because of the patient’s advanced age and massive rotator cuff tears with restricted ROM. Treatment combinations should be considered according to the severity of the rotator cuff tear, patient age, symptoms, and activity level.

Hemarthrosis of the shoulder is also rare. The association between rotator cuff tears and hemarthrosis has been reported by several investigators. Ishikawa et al. confirmed that the site of bleeding was the synovium, and based on the histological findings of the synovium taken during surgery, they speculated that in addition to anatomical and mechanical degeneration, biological reactions after phagocytosis may contribute to the bleeding [9]. Sano et al. reported a case of recurrent thrombosis in which minimal acromioplasty was performed after arthroscopic coagulation of the bleeding. They showed that the bleeding site was just medial to the greater tuberosity from the center of the cartilage defect in the humeral head and that the cartilage defect impinged on the medial margin of the acromion at 50° abduction. They concluded that the impingement due to osteophyte formation and instability may have contributed to the atrophy of the humeral head, and the intra-articular hemorrhage is caused by damage to the intraosseous blood vessels around the greater tuberosity [10]. Treatment options for repetitive hemarthrosis with massive rotator cuff tears have been proposed, including intra-articular steroid injections, synovectomy, rotator cuff repair, excisional arthroplasty, humeral head replacement, or total shoulder arthroplasty [10]. Fukuta et al. reported that hemarthrosis associated with rotator cuff tear arthropathy often requires total shoulder arthroplasty [4]. However, to the best of our knowledge, there are no such reports of hemarthrosis treated with RSA in the English literature. In this case, the bleeding point was not identified intraoperatively. The bone cyst was formed from the center of the head to the greater tuberosity, suggesting the presence of impingement in the area. It is assumed that the bleeding followed the mechanism described by Sano et al. [10]. A combination of treatments should be considered using the same approach employed for the ACJ cyst.

## Conclusions

Both ACJ cysts and hemarthrosis of the shoulder are rare conditions of massive rotator cuff tear and eventually lead to cuff tear arthropathy. Our report describes the first case of ACJ cyst and hemarthrosis of the shoulder that occurred simultaneously with rotator cuff tear arthropathy. RSA with excision of the cyst and distal clavicle was used to solve the three problems comprising rotator cuff tear arthropathy, ACJ cyst, and hemarthrosis of the shoulder with limited shoulder function. This report is very valuable in that it suggests that RSA is useful for both ACJ cysts and hemarthrosis of the shoulders associated with rotator cuff tear arthropathy.

## References

[1] Craig EV (1984). The geyser sign and torn rotator cuff: clinical significance and pathomechanics. Clin Orthop Relat Res.

[2] Shaarani SR, Mullett H (2014). Reverse total shoulder replacement with minimal ACJ excision arthroplasty for management of massive ACJ cyst - a case report. Open Orthop J.

[3] McCarty DJ, Swanson AB, Ehrhart RH (1994). Hemorrhagic rupture of the shoulder. J Rheumatol.

[4] Fukuta S, Miyatake K, Matsuura T, Sairyo K (2019). Two cases of spontaneous recurrent hemarthrosis of the shoulder with acromial erosion associated with impingement syndrome. Case Rep Orthop.

[5] Hiller AD, Miller JD, Zeller JL (2010). Acromioclavicular joint cyst formation. Clin Anat.

[6] Cvitanic O, Schimandle J, Cruse A, Minter J (1999). The acromioclavicular joint cyst: glenohumeral joint communication revealed by MR arthrography. J Comput Assist Tomogr.

[7] Postacchini F, Perugia D, Gumina S (1993). Acromioclavicular joint cyst associated with rotator cuff tear. A report of three cases. Clin Orthop Relat Res.

[8] Christodoulou KC, Kakagia DD, Galanis VG, Tsoucalas GI, Fiska AT (2021). Gigantic acromioclavicular joint cyst: presentation and mini review. J Shoulder Elbow Surg.

[9] Ishikawa K, Ohira T, Morisawa K (1988). Persistent hemarthrosis of the shoulder joint with a rotator-cuff tear in the elderly. Arch Orthop Trauma Surg.

[10] Sano H, Nakajo S (2004). Repeated hemarthrosis with massive rotator cuff tear. Arthroscopy.

